# Association between extracellular DNA levels, markers of inflammation and left ventricular mass index in children with chronic kidney disease

**DOI:** 10.1038/s41598-025-86857-4

**Published:** 2025-01-21

**Authors:** Ylva Tranæus Lindblad, Ľubomíra Tóthová, Peter Celec, Karolina Kublickiene, Peter Bárány, Milan Chromek

**Affiliations:** 1 Division of Pediatrics, Department of Clinical Science, Intervention and Technology, Karolinska Institutet, Stockholm, Sweden; 2 Institute of Molecular Biomedicine, Comenius University Medical School, Bratislava, Slovakia; 3 Division of Renal Medicine, Department of Clinical Science, Intervention and Technology, Karolinska Institutet, Stockholm, Sweden; 4 Pediatric Nephrology Unit, K86, Karolinska University Hospital Huddinge, 141 86, Stockholm, Sweden

**Keywords:** Chronic kidney disease, Extracellular DNA, Cell-free DNA, Cardiovascular disease, Kidney transplantation, Sterile inflammation, Chronic kidney disease, Predictive markers, Innate immunity, Paediatric research

## Abstract

Chronic kidney disease (CKD) is associated with chronic low-grade inflammation, but the primary factors triggering this inflammation remain unclear. Extracellular or cell-free DNA (exDNA) originates from virtually all tissues, being released during cell death, and stimulates the innate immune system. Our study was designed as an observational, cross-sectional cohort study of children with CKD (both before and after kidney transplantation) and controls to analyze associations between exDNA, markers of inflammation, and cardiovascular health. Extracellular DNA (total, nuclear, and mitochondrial) was analyzed in plasma using fluorometry and real-time PCR. We found that children with CKD after kidney transplantation had higher concentrations of total and nuclear extracellular DNA (total exDNA and nc_exDNA) in plasma compared to controls. In univariate analysis, levels of interleukin-6 (IL-6), antimicrobial peptide cathelicidin (LL-37), soluble vascular cell adhesion molecule-1 (VCAM-1) and left ventricular mass index (LVMI) were positively correlated with total exDNA and nc_exDNA concentrations. Multivariate analysis revealed LVMI as the only independent variable associated with high levels of both total exDNA and nc_exDNA. We believe that our results contribute new knowledge to the pathogenesis of CKD and its complications and may help identify new treatment targets.

## Introduction

Chronic kidney disease (CKD) significantly shortens life expectancy mainly due to high cardiovascular morbidity^1^. It is believed that CKD leads to a dysregulated ageing process associated with chronic low-grade inflammation^2^, high oxidative stress, impaired mitochondrial function, impaired calcium/phosphate/magnesium balance and dysbiosis of microbiota^3^. The consequences include vascular calcification and fibrosis, which are the hallmarks of cardiovascular disease (CVD)^4^.

Many uremic toxins, pro- and anti-inflammatory cytokines have been studied and found to be dysregulated in patients with CKD^5,6^. However, the primary factors that trigger and orchestrate chronic inflammation in CKD remain to be elucidated. In our study, we aimed to determine whether extracellular DNA (exDNA) could be one of the factors associated with inflammation and CVD in children with CKD.

Extracellular or cell-free DNA was discovered in human blood in 1948^7^. It originates from virtually all tissues as it is released during cell death^8^. Its major sources in plasma are precursors of red blood cells and immune system cells, especially neutrophils but its composition alters widely under different healthy conditions and under disease^9^. ExDNA consists of higher amount of nuclear (nc_exDNA) and lower amount of mitochondrial DNA (mt_exDNA). However, since the mt_exDNA involves much shorter fragments, the number of mt_exDNA copies is much higher compared to nc_exDNA^10^. ExDNA is not merely a waste product or marker of cell death, but it is also believed to play an important function in immune defense^11^. As a principal component of neutrophil extracellular traps, exDNA has a major role in anti-infection immunity^12^. If not cleaved rapidly, exDNA can induce and perpetuate inflammation by stimulating of innate immunity via Toll-like receptor 9 and other pattern-recognition receptors^13,14^. This seems to be one of the mechanisms that lead to cytokine storm and severe systemic inflammation during septicemia^15^. We hypothesize that a similar mechanism may apply to chronic sterile inflammation, as suggested for continuous metabolic syndrome in obesity^16^.

In middle-aged and older individuals, exDNA has been shown to be an independent predictor of all-cause mortality^17^. ExDNA levels were significantly higher in adult patients with CKD on hemodialysis as well as in adult patients with acute kidney injury^18^. Furthermore, high levels of exDNA in adult patients on hemodialysis were associated with high inflammation and high cardiovascular mortality^19^ with levels decreasing significantly after kidney transplantation^20^. In children, higher concentrations of exDNA were found in plasma of patients on peritoneal dialysis^21^, and donor-derived cell-free DNA was found in transplanted children with graft rejection^22^. As far as we know, the levels of total exDNA in children with pre-dialysis CKD and after kidney transplantation have not been studied. Importantly, exDNA is not only a marker and pathogenic factor in inflammatory states, but also presents a potential new treatment strategy. DNA-cleaving enzymes, DNases, are already routinely used in cystic fibrosis patients to cleave extracellular DNA in airway mucus^23,24^. Animal experiments suggested the potential systemic use of DNases in sepsis^15^ and hepatorenal injury^25^.

CVD is the main cause of mortality not only in adult patients with CKD, but the cardiovascular risk is already increased in pediatric CKD patients. Whilst the risk decreases after kidney transplantation, it remains higher in pediatric kidney transplant recipients compared to healthy peers^26^. The prevalence of preclinical cardiovascular changes, such as left ventricular hypertrophy (LVH) and left ventricular diastolic dysfunction are high in this population as shown by our group and others^27–30^.

The present study was designed to analyze associations between exDNA (total, nuclear and mitochondrial), inflammatory markers, and markers of cardiovascular health in children with CKD.

## Results

### Clinical characteristics

The clinical characteristics of 25 CKD and 40 kidney transplanted patients (CKD-T) as well as 10 controls are summarized in Table 1. The CKD patients were aged 7.8 years (range 0.8–18.8), while CKD-T patients were slightly older, with a mean age of 13.8 years (range 3.3–17.7). The controls did not differ in age from the patient groups (9.9 years, range 4.4–17.7). Regarding kidney function, all controls had a GFR > 90 mL/min/1.73 m^2^, while the majority of CKD patients were categorized as CKD stage 3–4, and most CKD-T patients had CKD stage 2–3.

**Table 1:** Clinical characteristics.

Clinical characteristics	Controls	CKD	CKD-T	p-value
	n = 10	n = 25	n = 40	
Age, year	9.9 [4.4–17.7]	7.8 [0.8–18.8]	13.8 [3.3–17.7]	**0.02c**
Males, n (%)	6 (60)	17 (68)	20 (50)	0.36
Duration of CKD, year		4.4 [0.8–14.5]	11.2 [1.8–17.4]	**0.0001**
Time after transplantation, year			5.6 [0.9–16.3]	
BMI, z-score	0.51 ± 1.3	0.26 ± 1.6	1.2 ± 1.4	**0.03c**
Obesity, n (%)	2 (20)	4 (16)	15 (37.5)	0.14
GFR, mL/min/1.73 m^2^	107 ± 11.9	32.8 ± 18.5	56.9 ± 20.6	** < 0.0001abc**
CKD stage 1 (GFR > 90 mL/min/1.73 m^2^), n (%)	10 (100)	0	3 (7.5)	
CKD stage 2 (GFR 60–89 mL/min/1.73 m^2^), n (%)	0	4 (16)	13 (32.5)	
CKD stage 3 (GFR 30–59 mL/min/1.73 m^2^), n (%)	0	7 (28)	23 (57.5)	
CKD stage 4 (GFR 15–29 mL/min/1.73 m^2^), n (%)	0	10 (40)	0	
CKD stage 5 (GFR < 15 mL/min/1.73 m^2^), n (%)	0	4 (16)	1 (2.5)	
Systolic blood pressure, z-score	0.51 ± 0.67	0.62 ± 1.3	0.74 ± 0.96	0.79
Diastolic blood pressure, z-score	0.23 ± 0.52	0.65 ± 0.96	0.44 ± 0.85	0.39
Hypertenstion (BP ≥ 95th percentile), n (%)	0	6 (24)	9 (22.5)	0.23
Albuminuria, n(%)	0	16 (64)	15 (37.5)	**0.002abc**
Medications				
Immunosuppressants, n (%)	0	2 (8)	40 (100)	** < 0.001bc**
Antihypertensives, n (%)	0	13 (52)	24 (60)	**0.003ab**

Data are presented as median [range] or mean ± standard deviation as appropriate. CKD chronic kidney disease, CKD-T kidney transplant recipients, BMI body mass index, GFR glomerular filtration rate, **a** Significant difference CKD versus controls, **b** Significant difference CKD-T versus controls, **c** Significant difference CKD-T versus CKD.

### Laboratory data

Laboratory data are presented in Table 2. Overall, while there was no difference in high-sensitive (hs)-CRP or pentraxin-3 between the groups, interleukin-6 (IL-6) was higher in CKD patients (2.3 [0.01–6.5] µg/L) and CKD-T patients (1.9 [0.22–25] µg/L) compared with controls (1.3 [0.57–2.5] µg/L), p = 0.03. White cell blood count (WBC) and antimicrobial peptide cathelicidin (LL-37) were highest among CKD-T patients, p = 0.02 and 0.0001 respectively.

**Table 2:** Laboratory data.

Laboratory data	Controls	CKD	CKD-T	p-value
Hemoglobin, g/L	132.2 ± 12.6	118.3 ± 12.3	121.9 ± 12.4	**0.01a**
Inflammatory markers				
hs-CRP, mg/L	0.54 [0.2–1.1]	0.43 [0.16–8.8]	0.35 [0.16–19.7]	0.78
Pentraxin-3, µg/L	1.1 [0.85–4.0]	1.3 [0.76–7.5]	1.4 [0.52–3.5]	0.87
IL-6, µg/L	1.3 [0.57–2.5]	2.3 [0.01–6.5]	1.9 [0.22–25]	**0.03ab**
WBC (× 10^9/^L)	5.5 [4.8–7.7}	6.0 [3.1–12.4]	7.4 [4.4–14.9]	**0.02bc**
LL-37, µg/L	27.7 [8.9–57.8]	35.4 [22.9–142.1]	80.7 [16.1–683.4]	**0.0001abc**
Vacular markers				
VCAM-1, µg/L	788 [452–992]	1017 [652–1430]	855 [643–1522]	**0.005ac**
ICAM-1, µg/L	259 ± 50.3	248 ± 67.0	224 ± 71.1	0.21
ExDNA				
Total exDNA, µg/L	7 [5.5–9.3]	7.7 [4.8–17.0]	8.9 [5.8–105]	**0.03bc**
Nc_exDNA, GE/mL	8677 [2219–21010]	13,215 [2676–41128]	15,012 [1383–441440]	**0.04b**
Mt_exDNA, GE/mL	382,847 [43293–1196058]	513,803 [192681–1.14e + 07]	470,852 [103757–3678934]	0.71

Data are presented as median [range] or mean ± standard deviation as appropriate. CKD chronic kidney disease, CKD-T kidney transplant recipients, hs-CRP high sensitivity-C-reactive protein, IL interleukin, WBC white blood cell count, VCAM-1 vascular cell adhesion molecule-1, ICAM-1 intercellular adhesion molecule-1, GE genomic equivalent, **a** Significant difference CKD versus controls, **b** Significant difference CKD-T versus controls, **c** Significant difference CKD-T versus CKD.

**Fig. 1:**
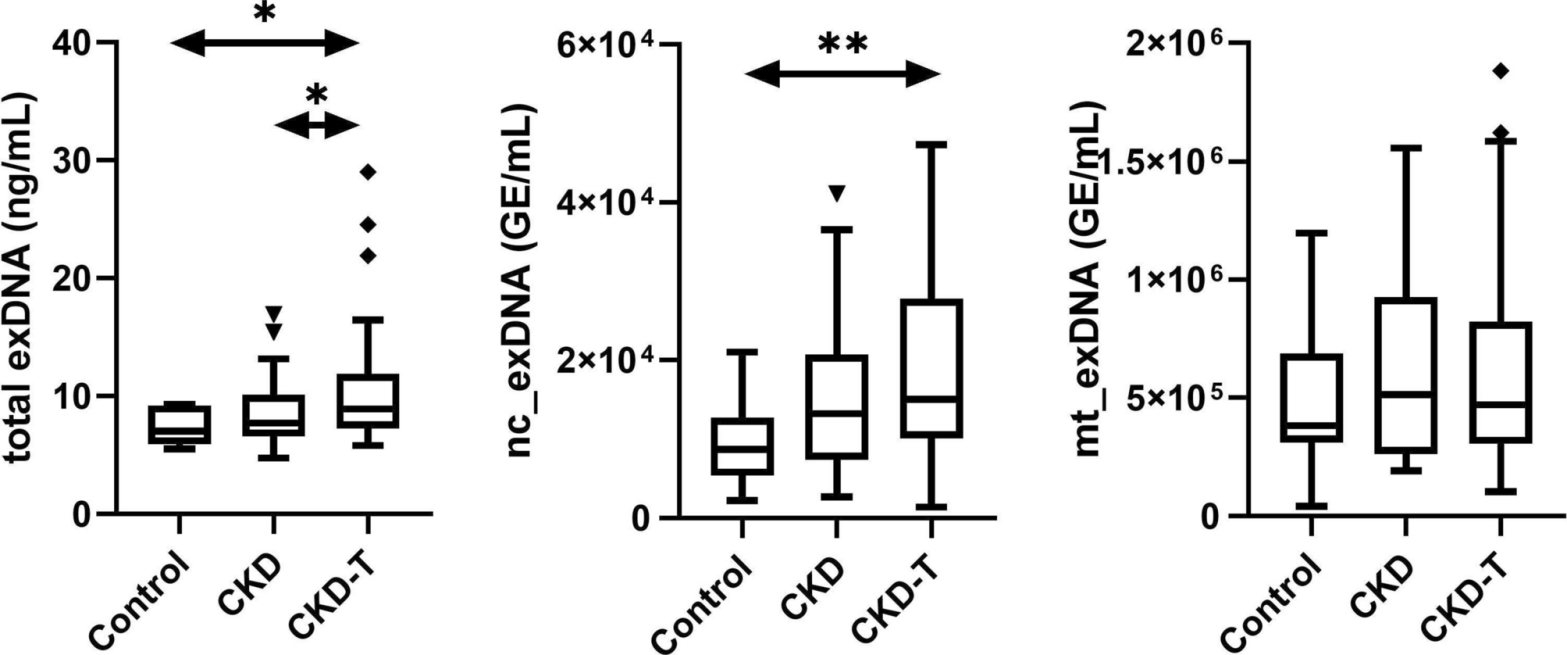
Plasma concentrations of total extracellular DNA (total exDNA), nuclear DNA (nc_exDNA), mitochondrial DNA (mt_exDNA) in controls (Control), chronic kidney disease patients (CKD) and patients after kidney transplantation (CKD-T). Total exDNA was higher among CKD-T patients compared with both CKD and controls and nc_exDNA was higher compared with controls. There was no difference in mt_exDNA between the groups. Data are presented as medians and Tukey box whiskers plots. GE genomic equivalent, * p < 0.05, ** p < 0.005.

Regarding exDNA, total exDNA was higher among CKD-T patients (8.9 [5.8–105] µg/L) compared with both CKD (7.7 [4.8–17] µg/L) and controls (7 [5.5–9.3] µg/L), p = 0.03. Nc_exDNA was also significantly higher among CKD-T patients compared with controls (p = 0.04), but there was no difference in mt_exDNA between the groups (Fig. 1).

### Echocardiographic data

Cardiac data are presented in Table 3. While all controls had normal LVMI, the prevalence of LVH was 19% in CKD and 26.9% in CKD-T patients. In addition, while no patient presented with left ventricular systolic dysfunction (EF < 50%), diastolic dysfunction defined as TDI (tissue Doppler imaging) lat eʹ z-score < 5th percentile was present in 15.8% of CKD and 15.4% of CKD-T patients*.*

**Table 3:** Echocardiografic data.

Echocardiographic data	Controls	CKD	CKD-T	p-value
LVMI, g/m^2.7^	30.0 ± 9.0	36.2 ± 7.8	39.9 ± 11.1	0.06
LVH, n(%)	0	4 (19)	7 (26.9]	0.29
Cc-TDI eʹ/aʹ	3.5 [2.4–7.1]	2.9 [1.8–6.3]	2.5 [1.6–4.4]	**0.02bc**
Cc-TDI lat eʹ, z-score	− 0.36 ± 0.59	− 0.59 ± 0.98	− 0.73 ± 0.98	0.60
Cc-TDI lat eʹ z-score < 5th percentile, n(%)	0	3 (15.8)	4 (15.4)	
PWD E/A	2.2 ± 0.58	1.8 ± 0.51	1.7 ± 0.40	**0.04b**
PWD E, z-score	− 0.82 ± 1.1	− 0.34 ± 0.73	− 0.10 ± 1.0	0.17
PWD E z-score < 5th percentile, n(%)	3 (37.5)	2 (9.5)	1 (3.8)	
PWD E/TDI eʹ	7.8 [7.1–9.2]	9.1 [6.8–13.3]	8.9 [6.5–17.5]	0.11
EF, %	68.9 ± 4.7	69.6 ± 4.8	71.7 ± 5.2	0.25
EF < 50%, n(%)	0	0	0	

Data are presented as median [range] or mean ± standard deviation as appropriate. CKD chronic kidney disease, CKD-T kidney transplant recipients, LVMI left ventricular mass index, LVH left ventricular hypertrophy, Cc-TDI color coded tissue doppler imaging, PWD pulse wave doppler, EF ejection fraction, **b** Significant difference CKD-T versus controls, **c** Significant difference CKD-T versus CKD.

### Correlations between exDNA, markers of inflammation and CVD

In univariate analyses of all study participants (Table 4, left), the inflammatory parameters WBC (p = 0.005), IL-6 (p = 0.04), LL-37 (p = 0.04) and soluble vascular adhesion molecule-1 (VCAM-1, p = 0.02) were positively significantly correlated to total exDNA. High LVMI (p = 0.004) and elevated BMI SDS (p = 0.007) were also significantly correlated to total exDNA. Similar pattern (apart from VCAM-1 and LL-37) was seen for nc_exDNA.

**Table 4:** Univariate associations (left) and associations in multivariate model (right).

Outcome	Independent variable	Univariate (Spearman)		Outcome	Independent variable	Multivariate model			
		β	p-value			β	p-value	Model-p	R^2^
Total exDNA	WBC	0.32	0.005	Log total exDNA	Log WBC	0.25	0.21	0.0008	0.47
	IL-6	0.24	0.04		Log IL-6	− 0.03	0.58		
	VCAM-1	0.26	0.02		Log VCAM-1	0.45	0.13		
	LL-37	0.24	0.04		Log LL-37	0.05	0.51		
	**LVMI**	0.39	0.004		**LVMI**	0.02	**0.005**		
	Patientgroup	0.31	0.007		Patientgroup	0.10	0.44		
	Time after Tx	− 0.34	0.03						
	BMI SDS	0.31	0.007		BMI SDS	− 0.01	0.87		
	Age	− 0.18	0.11		Age	− 0.006	0.68		
	Sex	0.08	0.52						
	GFR	− 0.04	0.74		GFR	0.002	0.31		
Nc_exDNA	WBC	0.26	0.03	Log nc_exDNA	Log WBC	0.17	0.68	0.04	0.26
	IL-6	0.25	0.03		Log IL-6	− 0.02	0.84		
	**LVMI**	0.35	0.009		**LVMI**	0.03	**0.03**		
	Patiengroup	0.28	0.02		Patiengroup	0.23	0.30		
	Time after Tx	− 0.46	0.003						
	BMI SDS	0.24	0.04		BMI SDS	− 0.03	0.80		
	Age	− 0.20	0.09		Age	− 0.02	0.58		
	Sex	0.04	0.71						
	GFR	− 0.08	0.47		GFR	0.001	0.82		
Mt_exDNA	IL-6	0.31	0.007	Log mt_exDNA	Log IL-6	0.12	0.32	0.03	0.25
	**LVMI**	0.34	0.01		**LVMI**	0.05	**0.007**		
	*LVH*	0.27	0.05						
	BMI SDS	0.29	0.01		BMI SDS	0.13	0.31		
	Age	− 0.02	0.90		Age	0.05	0.13		
	Sex	0.07	0.55						
	Patientgroup	0.04	0.75		Patientgroup	− 0.35	0.12		
	GFR	− 0.002	0.99		GFR	− 0.004	0.28		

WBC white blood cell count, IL interleukin, VCAM vascular cell adhesion molecule, BMI body mass index, LVMI left ventricular mass index, GFR glomerular filtration rate, LVH left ventricular hypertrophy.

In multivariate analyses (Table 4, right) a strong correlation between LVMI (β = 0.02, p = 0.005) and log total exDNA (model R^2^ = 0.47, p = 0.0008) was found. Similar correlation for LVMI were seen for log nc_exDNA (β = 0.03, p = 0.03), model R^2^ = 0.26, p = 0.04 and mt_exDNA (β = 0.05, p = 0.007), model R^2^ = 0.25, p = 0.03). However, none of the inflammatory markers measured were associated with log total exDNA, nc_exDNA or mt_exDNA in multivariate analyses.

## Discussion

Extracellular DNA is released from virtually all cells during cell death-whether through necrosis, apoptosis and NETosis^8^. Thus, it is not surprising that increased levels of exDNA have been found in conditions with significant cell turnover, such as infections, sterile inflammation, and mechanical cell damage due to trauma or dialysis^8,19,31^. In our study, we show that even children with CKD who are not on dialysis have high concentrations of extracellular DNA in their plasma.

In our univariate analysis, both total exDNA and nc_exDNA concentrations positively correlated with several inflammatory markers, including WBC, IL-6, LL-37, and VCAM-1. However, no such correlations were observed with hs-CRP or pentraxin-3, suggesting that the inflammatory response in CKD may be disease specific. Already in 1999, Stenvinkel et al. showed that CKD is characterized by chronic low-grade inflammation^2^. Since then, numerous inflammatory markers have been associated with CKD, particularly in adults. It is believed that inflammation in CKD increases oxidative stress, disrupts the calcium/phosphate/magnesium balance, and leads to vascular calcification and fibrosis that ultimately results in atherosclerosis. These processes contribute to premature vascular ageing and high cardiovascular morbidity^32^. Our study adds to this narrative by describing exDNA ich children with CKD.

Elevated exDNA levels could be the result of increased production, decreased clearance, or impaired degradation. Although transrenal exDNA has been found in urine^33^, its concentrations are negligible compared to those in plasma^34^, indicating that glomerular filtration rate (GFR) is unlikely to play a significant role in clearance of exDNA. Accordingly, we found no correlation between exDNA levels and GFR in our study. ExDNA is typically cleared by phagocytosis and/or extracellular degradation by DNA-cleaving enzymes (DNases). In animal model of acute kidney injury^35^, as well as in human studies on lupus nephritis^36^, lower DNase activity has been reported. However, DNase activity in CKD remains unexplored and warrants further investigation.

Importantly, exDNA is not only a marker but also a pathogenic factor and a potential therapeutic target for inflammation. If not rapidly cleared, exDNA acts as damage-associated molecular pattern (DAMP)^37^. Both nc_exDNA and mt_exDNA have been shown to stimulate innate immune system, though through different mechanisms. Mitochondrial DNA contains unmethylated CpG repeats, similar to bacterial DNA, which makes it particularly immunogenic. Mt_exDNA stimulates the immune system via Toll-like receptor 9 (TLR9)^13^. Nc_exDNA is recognized by cGMP-AMP synthase (cGAS), a newly discovered pattern recognition receptor^14,38^. Persistent high levels of either mt_exDNA or nc_exDNA may lead to chronic activation of the innate immune system, resulting in prolonged inflammation.

Interestingly, both total exDNA and nc_exDNA in our study positively correlated with cathelicidin (LL-37) levels. Cathelicidin is an antimicrobial peptide that plays a crucial role in immune defense, particularly on epithelial surfaces, in neutrophils, and macrophages^39^. LL-37 has an amphipathic structure with a strongly cationic region that binds to bacterial lipopolysaccharide as well as nucleic acids. This binding may hinder the autophagic recognition of free DNA and RNA, promoting inflammation in conditions as psoriasis and atherosclerosis^40,41^. We hypothesize that the production of cathelicidin may be a double-edged sword. While it protects against microbial pathogens^12,42,43^, its ability to stabilize exDNA could contribute to chronic inflammation and tissue damage in certain conditions^40^.

Both exDNA and cathelicidin are being tested as possible therapeutic targets. DNA-cleaving enzymes DNases are already used in patients with cystic fibrosis to cleave free DNA in airway mucus^23,24^. Furthermore, animal experiments showed promising results of systemic administration od DNases in models of sepsis and hepatorenal injury^15,25^. Cathelicidin production is stimulated by short-chain fatty acids, zinc and vitamin D, that are tested as a boost of anti-infection innate immunity^39^. If cathelicidin indeed plays a pole in perpetuating sterile inflammation and atherosclerosis, caution will be warranted in attempts to stimulate its production.

An unexpected finding in our study was the highest levels of total exDNA and nc_exDNA in transplanted patients. There are several possible explanations for this observation. Naturally, the patients after kidney transplantation differ from CKD patients before the transplantation in duration of CKD and in medications. Immunosuppressants could affect cell turnover and exDNA levels. Furthermore, the prevalence of hypertension was higher in transplanted patients in our cohort. Another intriguing question for the future studies is the origin of exDNA in transplant recipients. Previous studies have shown that transplant damage is often accompanied by increased levels of donor-derived exDNA in plasma. Analysis of exDNA could serve as a ‘liquid biopsy’ in the diagnosis of acute rejection, chronic allograft nephropathy, and adequacy of immunosuppressive treatment^44^.

Our study also analyzed several markers of cardiovascular health. Left ventricular mass index (LVMI), a marker of left ventricular hypertrophy and arterial stiffness was independently associated with total exDNA and nc_exDNA. This finding may have several explanations. ExDNA released during inflammation, for example, could contribute to increased arterial stiffness as indicated by higher LVMI. Alternatively, increased levels of exDNA could be the result of increased cell-death because of arterial stiffness.

We plan to continue following our cohort to assess whether high exDNA concentrations in childhood may have long-term implications for cardiovascular disease later in life.

Although our study is relatively small and single-centered, the significant differences motivate further investigation in larger multi-center studies. A major limitation in our study is the lack of DNases activity data in plasma due to limited availability of suitable samples.

In conclusion, this study demonstrates that children with CKD after kidney transplantation have elevated plasma levels of total exDNA and nc_exDNA. Both total exDNA and nc_exDNA correlated with markers of inflammation and LVMI. These findings suggest that exDNA may play a role in chronic inflammation and cardiovascular complications in CKD. We believe our results contribute new knowledge to the pathogenesis of CKD and its complications and could identify exDNA as a potential therapeutic target in the future.

## Methods

### Study population and design

The study was designed as an observational cross-sectional cohort study of children with CKD, either non-dialysis CKD stage 2–5 patients (CKD, n = 25) or kidney transplant recipients (CKD-T, n = 40). All patients were treated at the outpatient Pediatric Nephrology Unit at Astrid Lindgren Children´s Hospital, Karolinska University Hospital Huddinge in Sweden, with recruitment taking place between 2007 and 2008. Exclusion criteria were as follows: ongoing dialysis treatment, presence of congenital, structural, or primary myocardial disease, overt heart failure, HIV, Hepatitis C infection or an unstable clinical condition. A group of children with normal kidney function seen at the renal outpatient clinic for other diagnoses served as a reference-group (n = 10). The study cohort has been described in detail previously^27^.

### Clinical characteristics

Medical records were reviewed for etiology of kidney disease, duration of chronic kidney disease, time after kidney transplantation and records of medication. Clinical data were collected including values for height, weight, body mass index (BMI) and office blood pressure.

Standard deviation scores (z-scores) were calculated for systolic and diastolic blood pressures, as well as for BMI. Hypertension was defined as office systolic and/or diastolic blood pressure equal to or greater than the 95th percentile for age, sex, and height^45^ or current treatment with antihypertensive medication. Obesity was defined as BMI equal to or greater than the 95th percentile for age and sex^45^.

### Ethics approval

All procedures performed in studies involving human participants were in accordance with the ethics standards of the institutional and/or national research committee and with the 1964 Helsinki declaration and its later amendments or comparable ethical standards. Approval was granted by the Swedish Ethics Review Authority (Numbers 2005/977-31/3 and 2019–03498). All study participants and their parents or legal guardians approved participation in a written consent prior to the study.

### Laboratory data

Blood was drawn in a standardized manner during a clinical visit in the morning and following an overnight fast. Blood analysis of blood count and serum analyses of creatinine, cystatin C, calcium, phosphate, albumin, hs-CRP and WBC were performed in all study participants in the hospital lab using the automation platform Roche Cobas 8000 instrument module c701 in accordance with the manufacturer’s instructions. Plasma cathelicidin LL-37 was measured by ELISA kit from Hycult Biotech, Uden, the Netherlands. Pentraxin-3, IL-6, VCAM-1, ICAM ware analyzed by ELISA kits from R&D systems, Minneapolis, MN, USA. Early morning spot urine was collected to assess albuminuria, with a cut-off set at urinary albumin ≥ 20 mg/L. GFR was assessed by using iohexol or inulin/PAH clearances in all assessments but one where GFR was estimated from cystatin C^46^.

### Echocardiographic examination

Echocardiographic data were available for 21 CKD, 27 CKD-T patients and 7 controls. The echocardiographic examinations were carried out using a standard system (Vivid 7, GE VingMed Ultrasound, version 108.0.1, Horten, Norway). A two-dimensional guided M-mode measurement, conventional pulse wave Doppler (PWD), and color-coded tissue Doppler imaging (cc-TDI) were performed according to the American Society of Echocardiography (ASE) guidelines^47^. Left ventricular mass index (LVMI) was calculated (left ventricular mass/height^2.7^)^48^ as well as the presence of LVH^49^. Left ventricular diastolic function was evaluated with cc-TDI analyzing the peak myocardial velocities (cm/sec) during early (eʹ) and late (aʹ) diastole. The mean velocities of the septal and lateral margins of the mitral annulus were assessed for the eʹ/aʹ ratio, according to recommendations^49,50^. We used both raw data and calculated z-scores for cc-TDI é with cut-offs to define left ventricular diastolic dysfunction set at < 5th percentile^51^. An ejection fraction (EF) < 50% was used to define left ventricular systolic dysfunction. The detailed methods, longitudinal changes, and intra-observer variability of these echocardiographic analyses have been published^28^.

### Extracellular DNA isolation and quantification

Blood samples were centrifuged at 1600×*g* for 10 min prior to DNA isolation. Subsequently, the plasma supernatant was further centrifuged at 16,000×*g* for 10 min to remove apoptotic bodies. Samples were stored at − 20 °C until DNA isolation. DNA was isolated from 200 µl of plasma using a commercial kit (QIAamp DNA Mini Kit, Qiagen, Hilden, Germany) according to the manufacturer’s instructions. Fifty microliters of ultrapure water were used for the elution of DNA. DNA samples were stored at − 20 °C for later analysis.

The concentration of total exDNA was quantified by a fluorometric method using the high sensitivity dsDNA kit according to the manufacturer´s protocol (Qubit Fluorometer and Qubit dsDNA HS Assay Kit (Invitrogen, Carlsbad, CA, USA).

Subcellular origin of exDNA in isolates was determined by quantitative polymerase chain reaction (real-time PCR) on the QuantStudio 7 Flex Real-Time PCR Systems (Applied Biosystems, Waltham, MA, USA) using the SsoAdvanced Universal SYBR Green Supermix (Bio-Rad, Hercules, CA, USA). Primers encoding human beta-globin gene (F: 5ʹ-GCT TCT GAC ACA ACT GTG TTC-3ʹ, R: 5ʹ-CAC CAA CTT CAT CCA CGT TCA-3ʹ) and primers targeting D-loop (F: 5ʹ-CAT AAA AAC CCA ATC CAC ATC A-3ʹ, R: 5ʹ-GAC GGG TGG CTT TGG AGT-3ʹ) amplification were used to quantify nuclear DNA (nc_exDNA) and mitochondrial DNA (mt_exDNA), respectively. Thermal cycling conditions were used as follows: initiation 3 min at 98 °C, 40 × 15 s at 98 °C for denaturation, 30 s at 47 °C for annealing, 30 s at 60 °C for polymerization.

### Statistical analysis

Statistical analyses were performed using Stata (Statacorp, Texas, USA, version 16.0) and GraphPad Prism (La Jolla, CA, USA, version 9.5.0). Results are expressed as mean ± standard deviation and median [range]. One-way ANOVA or Kruskall-Wallis’s test were used for comparisons between groups. Post hoc testing using Dunn test or Bonferroni test were performed where appropriate.

Univariate correlations were analyzed using Spearman Rho, and for multivariate analyzes, ANOVA methods were used. Visually non-normally distributed variables plotted on histogram were tested for normality using Shapiro-Wilks test. Non-normally distributed variables were log-transformed before multivariate analyses. For comparison of categorical variables Chi-square analyses was used.

The primary outcomes in the multivariate models were log total exDNA, log nc_exDNA and log mt_exDNA. Variables with p-values < 0.05 in univariate models were tested in the multivariable models to fit the best model. Due to potential confounding, patient group (CKD, CKD-T or control), age (years), and GFR (ml/min/1.73 m^2^) were forced into the model. A p-value < 0.05 was considered statistically significant.

## Data Availability

The data underlying this article cannot be shared openly due to ethics restrictions and patient confidentiality. The pseudonymized data may however be shared by the corresponding author on reasonable request and after acquiring data use agreement.
